# Interventions for preventing obesity in children

**DOI:** 10.1590/1516-3180.20141322T2

**Published:** 2014-04-01

**Authors:** Elizabeth Waters, Andrea de Silva-Sanigorski, Belinda J. Burford, Tamara Brown, Karen J. Campbell, Yang Gao, Rebecca Armstrong, Lauren Prosser, Carolyn D. Summerbell

## Abstract

**BACKGROUND:**

Prevention of childhood obesity is an international public health priority given the significant impact of obesity on acute and chronic diseases, general health, development and well-being. The international evidence base for strategies that governments, communities and families can implement to prevent obesity, and promote health, has been accumulating but remains unclear.

**OBJECTIVE:**

This review primarily aims to update the previous Cochrane review of childhood obesity prevention research and determine the effectiveness of evaluated interventions intended to prevent obesity in children, assessed by change in Body Mass Index (BMI). Secondary aims were to examine the characteristics of the programs and strategies to answer the questions "What works for whom, why and for what cost?"

**METHODS:**

*Search methods:* The searches were re-run in CENTRAL, MEDLINE, EMBASE, PsychINFO and CINAHL in March 2010 and searched relevant websites. Non-English language papers were included and experts were contacted. *Selection criteria:* The review includes data from childhood obesity prevention studies that used a controlled study design (with or without randomisation). Studies were included if they evaluated interventions, policies or programs in place for twelve weeks or more. If studies were randomized at a cluster level, six clusters were required. *Data collection and analysis:* Two review authors independently extracted data and assessed the risk of bias of included studies. Data was extracted on intervention implementation, cost, equity and outcomes. Outcome measures were grouped according to whether they measured adiposity, physical activity (PA)-related behaviours or diet-related behaviours. Adverse outcomes were recorded. A meta-analysis was conducted using available BMI or standardized BMI (zBMI) score data with subgroup analysis by age group (0-5, 6-12, 13-18 years, corresponding to stages of developmental and childhood settings).

**MAIN RESULTS:**

This review includes 55 studies (an additional 36 studies found for this update). The majority of studies targeted children aged v 6-12 years. The meta-analysis included 37 studies of 27,946 children and demonstrated that programmes were effective at reducing adiposity, although not all individual interventions were effective, and there was a high level of observed heterogeneity (I2 = 82%). Overall, children in the intervention group had a standardised mean difference in adiposity (measured as BMI or zBMI) of -0.15kg/m^2^ (95% confidence interval (CI): -0.21 to -0.09). Intervention effects by age subgroups were -0.26kg/m^2^ (95% CI -0.53 to 0.00) (0- 5 years), - 0.15 kg/m^2^ (95% CI -0.23 to -0.08) (6-12 years), and -0.09 kg/m^2^ (95% CI -0.20 to 0.03) (13-18 years). Heterogeneity was apparent in all three age groups and could not explained by randomisation status or the type, duration or setting of the intervention. Only eight studies reported on adverse effects and no evidence of adverse outcomes such as unhealthy dieting practices, increased prevalence of underweight or body image sensitivities was found. Interventions did not appear to increase health inequalities although this was examined in fewer studies.

**AUTHORS' CONCLUSIONS:**

We found strong evidence to support beneficial effects of child obesity prevention programmes on BMI, particularly for programmes targeted to children aged six to 12 years. However, given the unexplained heterogeneity and the likelihood of small study bias, these findings must be interpreted cautiously. A broad range of programme components were used in these studies and whilst it is not possible to distinguish which of these components contributed most to the beneficial effects observed, our synthesis indicates the following to be promising policies and strategies:

- school curriculum that includes healthy eating, physical activity and body image;
- increased sessions for physical activity and the development of fundamental movement skills throughout the school week;
- improvements in nutritional quality of the food supply in schools;
- environments and cultural practices that support children eating healthier foods and being active throughout each day;
- support for teachers and other staff to implement health promotion strategies and activities (e.g. professional development, capacity building activities);
- parent support and home activities that encourage children to be more active, eat more nutritious foods and spend less time in screen based activities. However, study and evaluation designs need to be strengthened, and reporting extended to capture process and implementation factors, outcomes in relation to measures of equity, longer term outcomes, potential harms and costs. Childhood obesity prevention research must now move towards identifying how effective intervention components can be embedded within health, education and care systems and achieve long term sustainable impacts.

**This is the abstract of a Cochrane Review published in the Cochrane Database of Systematic Reviews (CDSR) 2011, issue 12, Art. No. CD001871. DOI:** 10.1002/14651858.CD001871.pub3 (http://onlinelibrary.wiley.com/doi/10.1002/14651858.CD001871.pub3/abstract). For full citation and authors details see reference 1.

**The full text is available from: **http://cochrane.bvsalud.org/doc.php?db=reviews&id=CD001871&lib=COC

## COMMENTS

Obesity during childhood and adolescence has become an issue of major concern over the last 10 years, around the world, regardless of race. This event has been named an epidemic and has much to do with profound changes not only in economic issues through higher per capita income, but also especially in dietary habits and lifestyle, in parallel with decreased physical activity due to a variety of reasons. Despite all the concerns about overweight/obesity and the advent of numerous diets for weight reduction, it appears that most of them have been ineffective in reducing weight. Thus, this review is very timely, particularly given the importance of evaluating the effectiveness of various intervention programs for obese children. 

Worldwide experience in this regard has been very extensive, involving governments, communities and families. Nonetheless, at the present time, there is no good evidence about the best strategy for health promotion relating to weight loss.

The first objective of this review was to determine the effectiveness of intervention programs for preventing obesity in children, as assessed by the body mass index (BMI). This form of evaluation of programs has questionable sensitivity among children, whose BMI is not a parameter that reflects weight loss fairly, since only the influence of growth itself can interfere with BMI, even if there is only a slight weight reduction. This becomes more important during puberty. Likewise, children and especially teenagers who are committed towards impact sports that have important influences on body composition will also present divergent results. 

The second objective can be summarized as a review of the characteristics of the programs and strategies that fit these patients, as well as their costs and benefits.

In accordance with the review criteria, all studies with control groups were included. However, not all studies reviewed were randomized, which may have facilitated entry in the group of children and or families who were already motivated to lose weight, which may have interfered with the results obtained.

The authors included 37 studies in the review, corresponding to 27,496 children aged 6 to 12 years, and they concluded that the programs were effective for reducing adiposity, although not all interventions have reached good results. Furthermore, there was great heterogeneity in the results found, which could not be adequately explained by the review authors, and they suggested that caution is needed in interpreting the results. Some of the issues that were pointed out here, along with the study population itself, and the possibility of bias would probably explain this heterogeneity, including the need for better assessment of the behavior of randomized trials, in comparison with non-randomized trials. Evaluation of the percentage of the children who did not lose weight but had stabilized, or those who may have gained weight, would perhaps have been helpful for our understanding of the results obtained. Moreover, it also needs to be borne in mind that the clinical approach towards obese patients should always be individualized, especially in relation to children who are exposed to different kinds of influences, from families, peers, school, the environment and so on.

This review was also unable to identify which aspects of the programs have in fact contributed to the slight weight loss. Nevertheless, in the discussion, which is very well written, the authors stressed some very important issues: the need to improve environmental conditions and cultural practices so as to emphasize healthy food intake; the need for a curriculum, including notions of healthy eating, physical activity and body image; and educational support for teachers in relation to health promotion activities. However, it is important to stress that, without adequate participation and awareness among families, it will be difficult for any program to succeed. Given that these authors also suggested that increased physical activity during the school week is important, it must also be said that in certain areas and countries, it is very difficult to implement these activities due to the lack of public policies. 

This review has not added any new facts and, in effect, does not indicate what type of intervention promotes better outcomes. Its conclusions are well known in approaches towards obese children that have already become part of routine care. Perhaps the time available for action in the studies included was very short, such as the minimum of 12 weeks, which may have been insufficient to promote changes in behavior. On the other hand, this underlines the need to establish public policies that allow teachers and educators to teach about nutrition, increased physical activity in schools and creation of community spaces for practicing exercises, with full participation by families. Perhaps, rather than focusing only on weight, it is more important to work on adherence to healthier eating habits and exercise, not only towards weight loss but also towards health promotion. Assessment of body composition is also becoming more important than BMI seen in isolation. Future evaluations need to focus on following up the evolution of weight after the intervention and on maintaining the knowledge acquired over the long term, because only through education will it be possible to prevent obesity and its comorbidities, as a lifetime program. 

**Angela Maria Spinola e Castro**. MD, PhD. Associate Professor of the Department of Pediatrics, Escola Paulista de Medicina, Universidade Federal de São Paulo (EPM-Unifesp); Head of Pediatric Endocrinology and Chairman of the Department of Endocrinology, Associação Paulista de Medicina (APM), São Paulo, Brazil.
